# Predictors of survival outcomes in native sub Saharan black men newly diagnosed with metastatic prostate cancer

**DOI:** 10.1186/s12894-017-0228-0

**Published:** 2017-05-30

**Authors:** Jibril Oyekunle Bello

**Affiliations:** Department of Surgery, University of Ilorin Teaching Hospital, Ilorin, Nigeria

**Keywords:** Prostate cancer, Androgen deprivation therapy, PSA kinetics, PSA nadir, Black men, Sub Saharan Africa

## Abstract

**Background:**

Though it is well established that black men are at higher risk of prostate cancer (PCa) very little is known about the disease in native sub Saharan black men. Newly diagnosed metastatic PCa patients treated with primary androgen deprivation therapy were identified and predictors of progression-free survival (PFS) assessed.

**Methods:**

Patients diagnosed with metastatic PCa between 2010 and 2015 in a sub Saharan black population were included in the study. Primary outcome measure was PFS defined as time from primary androgen deprivation therapy to clinical progression or death. Demographic, clinical and PSA kinetic variables were evaluated for their prognostic power using Cox proportional hazard regression models.

**Results:**

Seventy-nine patients met the eligibility criteria and were analyzed. Median age, median overall survival and PFS was 69 years, 40 months and 27 months respectively. A PSA nadir >4 ng/mL was found to predict an earlier clinical progression. Median PFS was shorter in those with PSA nadir >4 ng/mL (15 months) compared to those with PSA nadir ≤4 ng/mL (29 months); log rank *p* value = 0.003.

**Conclusions:**

The PSA nadir achieved following primary androgen deprivation therapy predicts progression-free survival in sub Saharan black men newly diagnosed with metastatic PCa. PSA nadir >4 ng/mL was found to be associated with a more rapid clinical progression.

## Background

Although the exact burden of Prostate cancer (PCa) in sub Saharan Africa remains largely unknown [1]; the region is home to the largest populations of black men, a well-established high risk group for PCa. Unfortunately, despite this, very little is known about the basic and clinical aspects of the disease in these native black men. This is probably due to weak health systems, poor cancer registries and a hostile research environment with poor funding [2]. In the few studies that are available, findings have supported the widely-held view that significantly higher proportion of men in the region present late with advanced disease compared to most other regions of the world [3]. This may be attributable to a near lack of any established systematic screening practices in the region during the early PSA era when PCa screening was encouraged up to current times when population screening has become controversial. In our practice, well over half of newly diagnosed patients have locally advanced or metastatic disease.

Primary androgen deprivation therapy (PADT) is the most widely used and most potent systemic treatment for newly diagnosed hormone sensitive metastatic PCa. It is probably the most common form of treatment for PCa received by black men of sub Saharan Africa largely due to the much higher proportion of men with advanced disease. PADT is often in the form of bilateral orchiectomy with or without antiandrogens for the majority of these men mainly due to cost considerations as health insurance coverage are generally low. Despite PADT, most men progress biochemically and clinically eventually to castrate resistant disease and or death. Wide variability in clinical progression of metastatic PCa has been observed and though certain demographic, PSA kinetic and histopathological markers have been reported to be associated with higher mortality risk, most of the studies were done with moderate to fair representation of black men generally but scarcely inclusive of any native sub Saharan black men [4–7]. There is thus a need to determine surrogate markers of aggressive progressive disease and impaired survival in our very peculiar and under studied high risk population. This retrospective study queried the clinical records of post-PADT native black men newly diagnosed with metastatic disease to assess progression-free survival (PFS) and overall survival (OS) and determine possible early surrogate markers of rapidly progressive disease. Although OS is the standard endpoint in survival analysis, PFS is also a relevant endpoint in the setting of metastatic PCa as patients often eventually die of disease progression.

## Methods

This cohort was generated from clinical records of newly diagnosed black men with metastatic PCa who presented for care at a tertiary care hospital in North Central Nigeria, West Africa between June 2010 and July 2015. To be eligible for inclusion in the study, patients must have had metastatic bone disease confirmed with radiological investigations and subsequently agreed to androgen deprivation therapy (orchiectomy or luteinizing hormone-releasing hormone with or without anti androgens). Patients would also have had a pretreatment PSA and at least 2 post treatment PSA readings done less than 12 months’ post androgen deprivation therapy with at least one assay between the third and eighth month. Patients who had prior primary surgery (prostatectomy) or radiotherapy were excluded from the study. Demographic and clinical information retrieved include age, use of Non-steroidal anti-inflammatory drugs (NSAID), pretreatment PSA, PSA decline, Post treatment PSA nadir and Gleason score. Post treatment PSA nadir was defined as the lowest PSA reported during the first 12 months following androgen deprivation therapy. PSA decline was defined as the relative change of PSA nadir from baseline (pretreatment PSA). This study’s primary outcome measure was progression-free survival (PFS). PFS was defined as time from commencement of androgen deprivation therapy to clinical progression or censure. Clinical progression was defined as a composite outcome of the occurrence of new metastases or skeletal related events or death from PCa. Categorical and continuous variables were summarized using percentages and medians (interquartile range) respectively. Univariate logistic and multivariate Cox proportional hazard models with backward elimination were used to explore the impact of potential predictors. PFS was estimated using the Kaplan-Meier method with significance determined by log rank test. All analyses were performed with SPSS software, version 22.0 (SPSS Inc), *p* value <0.05 was considered to be statistically significant. Institutional ethics board approval was obtained for the study.

## Results

**Fig. 1:**
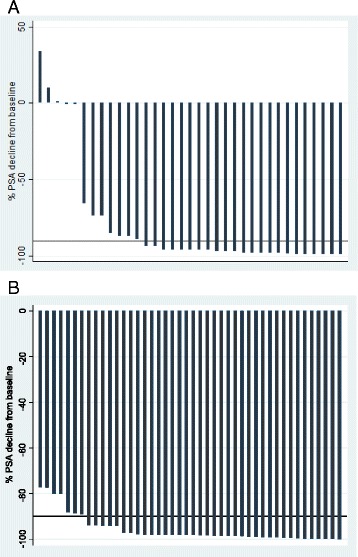
Waterfall plots of relative PSA decline from baseline for patients with (**a**) and without (**b**) clinical progression during study follow-up (median 15 months). A decline of ≥90% was seen in 23 (65.7%) and 37 (84.1%) patients in (**a**) and (**b**) respectively. This was not statistically significant (*p* = 0.06). The bold reference line represents PSA decline of 90%

**Fig. 2:**
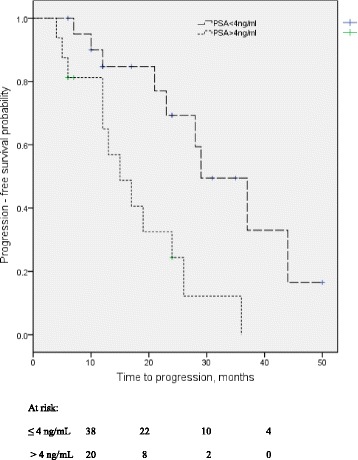
Progression-free survival probability of patients with metastatic prostate cancer following primary androgen deprivation therapy. Kaplan-Meier analysis showed significant differences between patients with PSA nadir ≤4 ng/mL Vs > 4 ng/mL, log-rank *p*-value = 0.003

Seventy-nine patients met the eligibility criteria and were analyzed. The baseline characteristics of the patients are shown in Table 1. The median age and pretreatment PSA at diagnosis were 69 years (IQR: 65 – 73 years) and 94 ng/mL (IQR: 50 – 124 ng/mL) respectively. Majority of the men 65(82.3%) had orchiectomy with or without anti androgens. The median PSA nadir was 4 ng/mL (IQR: 1.0 – 9.0 ng/mL) and 60 (75.9%) patients achieved a PSA decline of ≥ 90%. The median time from PADT to PSA nadir was 7 months (IQR: 5-8). The median OS and PFS was 40.3 months (95%CI 24.3-49.7 months) and 26.8 months (95% 19.1 – 28.9 months) respectively. After a median follow-up of 15 months (IQR: 12 – 24 months) clinical progression was documented in 35 (44.3%) patients and of these 19 (23.8%) died. Prognosticators of clinical progression found on univariate analysis (Table 2) were analgesic consumption (NSAID use) and PSA nadir. Pretreatment PSA and PSA decline were not significant prognosticators; waterfall plots (Fig. 1) of PSA decline for patients with (A) and without (B) clinical progression during study follow-up show that a decline of ≥ 90% was not significantly different in both groups (*p* = 0.06). PSA nadir was dichotomized at median (4 ng/mL) and this was also found to be predictive. On multivariate analysis (Table 2), only PSA nadir remained as statistically significant predictor with reduced PFS apparent in patients with PSA nadir >4 ng/mL (HR = 2.85, 95% CI: 1.06-7.67). Kaplan Meier plot of PFS by PSA nadir (Fig. 2) illustrated the differences between the two survival distributions (PSA nadir ≤4 ng/mL versus PSA nadir >4 ng/mL) with the median PFS for patients with PSA nadir ≤4 ng/mL of 29 months (95%CI 19.3 – 38.7) significantly longer than that of those with PSA nadir >4 ng/mL which was found to be 15 months (95%CI 8.3 – 21.7), log-rank *p*-value = 0.003.

**Table 1:** Baseline characteristics of study cohort (*n* = 79)

Variables	*n* (%)	median^a^(IQR)
Age		69.0 (65.0 – 73.0)
Place of residence		
Urban	56 (70.9)	
Rural	23 (29.1)	
Initial Clinical presentation		
LUTS	69 (87.3)	
^b^NSAID use	51 (64.6)	
Anaemia (<10 g/dL)	25 (31.6)	
PADT received		
Orchiectomy	65(82.3)	
LH-RH analogues	14 (17.7)	
PSA kinetics		
Pre-treatment PSA(ng/mL)		94.0 (50.0 – 124.0)
PSA nadir (ng/mL)		4.0 (1.0 – 9.0)
PSA decline		
≥ 90%	60 (75.9)	
< 90%	19 (24.1)	
Biopsy Gleason score		
3 + 3	24 (30.4)	
3 + 4	10 (12.7)	
4 + 3	20 (25.3)	
≥ 8	25 (31.7)	

^a^Interquartile range, ^b^Non-steroidal anti-inflammatory drugs

**Table 2:** Cox proportional hazards models for progression-free survival following primary androgen deprivation therapy

Variables	Univariate			Multivariate		
	HR	95% CI	p	HR	95% CI	p
Age	1.01	0.96-1.06	0.706			
Urban residence	1.44	0.59-3.48	0.421			
LUTS	2.15	0.70-6.57	0.181			
^a^NSAID use	3.80	1.43-10.10	0.007	3.40	0.74-15.66	0.117
Anaemia	1.89	0.77-4.64	0.162			
Pretreatment PSA	1.00	0.99-1.01	0.913			
PSA decline						
< 90%	1.00 (Reference)					
≥ 90%	0.36	0.13-1.10	0.063			
^a^PSA nadir						
≤ 4 ng/mL	1.00 (Reference)					
> 4 ng/mL	3.86	1.49-10.04	0.006	2.85	1.06-7.67	0.038
Biopsy Gleason score						
6	1.00 (Reference)					
7	0.82	0.35-1.91	0.648			
8-10	1.26	0.56-2.80	0.579			

^a^significant at univariate analysis and carried onward to multivariate analysis

## Discussion

In sub Saharan Africa, urologists are often faced with the dilemma of how to interpret prostate cancer guidelines mostly derived with suboptimal representation of black men (and scarcely any native sub-Saharan African men). This problem is compounded by the dearth of research on the basic and clinical aspects of PCa from sub Saharan Africa and the weak healthcare system of the region which offers fewer options in the treatment of PCa. The resulting knowledge gap is particularly remarkable taking to consideration the high-risk status of the population and the fact that a large majority of native African black men currently present with aggressive disease and at a late stage with metastases [3, 8, 9]. As observed in other populations, wide variability exists in the clinical course of metastatic PCa in sub Saharan African men. This study of a cohort of sub Saharan African men thus examined the role of some demographic and clinical (including PSA kinetics) variables in predicting clinical progression following PADT.

Median age of our study cohort was 69 years which is similar to that found in similarly studied sub Saharan African populations [3, 8, 9]. There are no available studies of sub Saharan black men that evaluated the relationship between age and survival for comparison with this study. However, a study carried out in the United States, in which African Americans formed 10% of the study population found that the prognostic power of age though significant at univariate analysis was lost at multivariate analysis that controlled for treatment modality [4]. This suggests that where treatments are similar survival outcomes would probably not be different across age groups. Another study from the same region in which blacks were about one-sixth of the study population found that younger men, who were more likely to be black, were at higher risk of all cause and cancer specific death [5]. This could be interpreted to mean that young black men were more likely to die than other groups. This current study done exclusively in native black men of sub Saharan Africa found no such relationship though it investigated the broader outcome of PFS; and it corroborates Koff et al. findings of no relationships between age, race and PFS [10]. NSAID use was found to be associated with decreased PFS at univariate analysis in this study but this association was lost when PSA nadir was included in multivariate analysis. About two-thirds of the patients studied required analgesia for pains related to metastasis. No association was found between Gleason score and PFS in this study.

While pretreatment PSA, PSA velocity and Gleason score have been found to be important predictors of response to therapy and survival in the setting of localized disease [11, 12]; alkaline phosphatase, PSA nadir and time to PSA nadir, PSA half-life have been reported to predict survival following PADT for advanced or metastatic disease [6, 7, 10, 13–17]. The importance of these variables has never been investigated in a native African black population. While many studies have reported that lower PSA nadir reflects a good prognosis with slower clinical progression, there are no agreed values of optimum PSA nadir above which prognosis worsens. Reported values include 0.1 ng/mL, 0.2 ng/mL, 2 ng/mL and 4 ng/mL [7, 10, 13, 15]; these different values may be attributable to the dissimilar cohorts studied. Some cohorts included patients who had prior treatment with curative intent (prostatectomy or radiotherapy), some included only newly diagnosed metastatic PCa patients while others included a mix of patients with localized, locally advanced or metastatic disease. This current study includes only newly diagnosed metastatic PCa patients and found that a PSA nadir >4 ng/mL was associated with earlier clinical progression; this is similar to findings of Hussain et al. in their study cohort of metastatic PCa patients who were treated with 7-month “induction” course of Goserelin and bicalutamide [7]. Hussain et al. assessed for OS and found a median OS of 13 months for patients with PSA nadir of more than 4 ng/mL which was significantly differently from those in their cohort with PSA less than 4 ng/mL or less than 0.2 ng/mL [7]. In contrast, the current study assessed PFS (clinical progression and or death as endpoints) and found a PFS of 15 months in cohort members with PSA nadir >4 ng/mL which was significantly less than the PFS of 29 months found in those with PSA nadir ≤4 ng/mL. Unlike PSA nadir, evaluations of the prognostic value of time to PSA nadir (TTN) have produced inconsistent results [6, 14, 16–18]. Both shorter and longer TTN have been found to be associated with worse outcomes. These varied results may be due to dissimilar cohorts or inappropriate statistical analysis; more frequent PSA measurements done at similar intervals would be required for appropriate analysis of TTN. This was unavailable in the current study and TTN was not analyzed.

There are some important limitations to this study. Admittedly it is a retrospective study and cohort size is not large. Additionally, predictors of PFS and not OS were assessed because of the relatively few events (deaths) that occurred during the study period. Despite these limitations, this is the first study to report on the predictors of clinical progression in native sub Saharan African black men with metastatic PCa. Findings from this study may help identify patients likely to develop early clinical progression and these patients may benefit from early commencement of other therapies aimed at delaying clinical progression and prolonging survival. In our resource-limited setting, patients commonly receive chemohormonal therapy with docetaxel added to PADT following clinical progression to castrate-resistant PCa. The high costs associated with newer drugs, abiraterone and enzalutamide, make them out-of-reach for patients in our low- and middle- income region where payments for health care are mainly out-of-pocket and health insurance coverage is low.

## Conclusions

The PSA nadir achieved following primary androgen deprivation therapy predicts progression-free survival in sub Saharan black men newly diagnosed with metastatic PCa. PSA nadir >4 ng/mL was found to be associated with a more rapid clinical progression.

## Abbreviations

NSAID: Non-Steroidal anti-inflammatory drugs
OS: Overall survival
PADT: Primary androgen deprivation therapy
PCa: Prostate cancer
PFS: Progression-free survival
PSA: Prostate-specific antigen
TTN: Time to PSA nadir
